# The validation of Chinese version of workplace PERMA-profiler and the association between workplace well-being and fatigue

**DOI:** 10.1186/s12889-024-18194-6

**Published:** 2024-03-06

**Authors:** Chen–Cheng Yang, Hsiang-Tai Chen, Kuei-Hau Luo, Kazuhiro Watanabe, Hung-Yi Chuang, Chih-Wei Wu, Chia–Yen Dai, Chao-Hung Kuo, Norito Kawakami

**Affiliations:** 1 Graduate Institute of Medicine, College of Medicine, Kaohsiung Medical University, 807 Kaohsiung City, Taiwan; 2 Department of Occupational and Environmental Medicine, Kaohsiung Municipal Siaogang Hospital, Kaohsiung Medical University, 812 Kaohsiung City, Taiwan; 3 Department of Occupational and Environmental Medicine, Kaohsiung Medical University Hospital, Kaohsiung Medical University, 807 Kaohsiung City, Taiwan; 4 College of Health and Medicine, University of Tasmania, 7000 Hobart, TAS Australia; 5 Department of Public Health, Kitasato University School of Medicine, 1-15-1 Kitazato, Minami-ku, 252-0374 Sagamihara, Japan; 6 Department of Surgery, Shin Kong Wu Ho Su Memorial Hospital, 111 Taipei City, Taiwan; 7 Department of Internal Medicine, Kaohsiung Medical University Hospital, Kaohsiung Medical University, 807 Kaohsiung City, Taiwan; 8 Department of Internal Medicine, Kaohsiung Municipal Siaogang Hospital, Kaohsiung Medical University, 812 Kaohsiung City, Taiwan; 9 Department of Digital Mental Health, Graduate School of Medicine, The University of Tokyo, Bunkyo-ku, 113-0033 Tokyo, Japan

**Keywords:** Checklist individual strength, Fatigue, Workplace PERMA-profiler, Well-being, Occupational medicine, Positive psychology

## Abstract

**Background:**

Well-being is an important issue in workplace. One of these assessment tools of well-being, Workplace PERMA Profiler, is based on Seligman’s five dimensions well-being. Prolonged fatigue may last for a long time, leading a great impact on both employees and enterprises. However, rare studies about the association between well-being and fatigue had been investigated. Our aim is to establish the Chinese version Profiler, and to discovery the association between workplace well-being and fatigue.

**Methods:**

The Chinese version was established according to International Society of Pharmacoeconomics and Outcomes Research (ISPOR) task force guidelines. In the study, researchers employed simple random sampling by approaching individuals undergoing health checkups or receiving workplace health services, inviting them to participate in a questionnaire-based interview. Prolonged Fatigue was evaluated by Checklist Individual Strength (CIS). The reliability was evaluated by Cronbach’s alphas, Intra-class Correlation Coefficients (ICCs), and measurement errors. Moreover, confirmatory factor analysis and correlational analyses were assessed for the validity.

**Results:**

The analyses included 312 Chinese workers. Cronbach’s alphas of the Chinese version ranged from 0.69 to 0.93, while the ICC ranged from 0.70 to 0.92. The 5-factor model of confirmatory factor analysis revealed a nearly appropriate fit (χ^2^ (82) = 346.560, Comparative Fit Index [CFI] = 0.887, Tucker-Lewis Index [TLI] = 0.855, Root Mean Square Error of Approximation [RMSEA] = 0.114, Standardized Root Mean Square Residual [SRMR] = 0.060). Moreover, the CIS and its four dimensions were significantly and negatively associated with the Positive Emotion, while they are positively associated with Engagement dimension except CIS-Motivation dimension.

**Conclusion:**

The Chinese version Workplace PERMA-Profiler indicate nice reliability and validity. Furthermore, all CIS dimensions were negatively influenced by Positive Emotion, while commonly positively associated with Engagement.

**Supplementary Information:**

The online version contains supplementary material available at 10.1186/s12889-024-18194-6.

## Background

### Well-being

Well-being refers to a feeling of good and functional experience. A famous social psychologist, Marie Jahoda, pointed out mental health should be not merely the lack of mental illness, which is an innovative idea of positive psychology [1].

There are usually two aspects of well-being: the hedonic aspect and the eudaimonic (meaning of life) aspect. The hedonic aspect is regarded as positive emotion toward human experience. In the condition, well-being is often considered as the best balance between positive emotion and negative emotions, and satisfaction of life [2, 3]. In contrast, the eudaimonic aspect is constructed on virtuous action and self- realization. The feeling of well-being is constructed on one’s ability to exert one’s strength and abilities [4]. Although hedonic does not define well-being by a single formula, it focuses on the subjective construction of the individual, the point of view of eudaimonic is more theoretical. The concept of eudaimonic considers it is not appropriate to define well-being based on only concerned with emotional satisfaction but ignores the important aspect of function. However, well-being should not be evaluated merely on personal assessment. Recently, investigations emphasis on redefining well-being as the mixture of hedonic and eudaimonic aspects, which combines these two ideas gradually [5, 6].

In 2011, Seligman integrated hedonic and eudaimonic aspects, and brought the theory of well-being, or so-called PERMA model. His theory declared that well-being is not just a lack of negative emotion, as well as consists of PERMA model [7]. PERMA model is assembled by five elements: Positive Emotion, Engagement, Relationships, Meaning, and Accomplishment [7]. As a relatively new theory, there has not been a standardized brief verification measurement for evaluating of the five proportions of PERMA models. Several scales have been developed, such as Huppert’s flourishing item [8], and Su’s Brief Inventory of Thriving [9], are consisted of the five proportions, but each proportion only contains one or two items. In 2013, the tool developed by Huppert and So, consisted the 5 proportions of PERMA, as well as emotional stability, optimism, resilience, self-esteem, and vitality, which one item represents each dimension [8]. Su’s 54-item Comprehensive Inventory of Thriving contains a series proportions such as learning, self-worth, lack of autonomy, and optimism besides PERMA structure [9].

In 2016, Butler and Kern developed and validated a scale for measuring the PERMA model, the PERMA-Profiler. After consolidating the questionnaire into 15 core items, they validated in two study groups, analyzed the structure relationship among factors, and then added 8 items, which finally becoming a version consisted of 23 items [10].

### Workplace well-being and workplace PERMA-profile

The definition of workplace well-being should be solved by development and operation of the established scale [11]. Most well-being models have corresponding scales. For example, Kern developed PERMA-profiler for monitor well-being status. This scale can compare with previous scale, to cooperate the proportion of well-being [12]. However, workplace is difference from daily life, the occupational version PERMA profiler was established by Kern in 2016, adjusting the questionnaire to evaluate the workplace appropriately [13]. Comparing with general version, workplace version included 15 items for the five proportions (Positive Emotion, Engagement, Relationship, Meaning, and Accomplishment), one item of Happiness, three items of Negative Emotion, three items of Health, and one item of Loneliness. To our knowledge, no study about well-being in workplace, especially by workplace PERMA model had been conducted in Taiwan [13].

### Fatigue

Fatigue is a common complaint and has huge impacts on health owning to its excessive influences on one’s life quality. Although fatigue is believed a predict factor for illnesses, it is difficult to be defined and measured. Appels et al. reported a strong association between fatigue and mortality [14]. De Croon et al. showed that the requirement of retrieval can be a prediction of over 14 days long term sick leave in the future [15]. Extensive research indicates that fatigue is closely linked to diminished cognitive function, occupational accidents, metabolic disorders, reproductive issues, certain cancers, and increased mortality rates, along with mental, gastrointestinal, neurological, and chronic pain conditions [16, 17]. Despite the well-documented consequences of fatigue and the importance of strategies to mitigate its risks for the health and productivity of employees in safety-critical roles, research on the link between well-being and fatigue in the workplace remains scarce. Fatigue can be classified into acute and prolonged fatigue according to the period length [18]. Acute fatigue is a normal sensation during specific job content, and it might be vanished when job transfer or special compensation strategy (such as working in a slower temple or taking rest for a period) [18]. In contrast, prolonged fatigue is the cumulative result. It probably could be happened because someone exposes to one or multiple stress factors and does not have enough recovery time. Prolonged fatigue is not caused by specific task. The compensation mechanism in a short time is invalid and could not reverses prolonged fatigue. Prolonged fatigue is a weakness status that may cause negative effect on life [18].

There is lack of a gold standard of objective measurement for prolonged fatigue. Current objective evaluation focuses on the physical process or presentation, for testing the number of errors or recording the reaction time [19]. Personal evaluation method for fatigue includes conducting interview, writing diary, or using questionnaire [20, 21]. For convenience, questionnaire is an appropriate method for large scale study. Several scales were published for evaluating fatigue status in workplace. Fatigue scale (in abbreviation of FS) is a 5-point Likert scale, which divide 4 items for psychological fatigue and 7 items for physical fatigue [22]. Vercoulen et al. developed Checklist Individual Strength Questionnaire (in abbreviation of CIS), being reliable for evaluating prolonged fatigue and mostly used over the world [23]. It is consisted of 20 items and participants must fulfill the 7-point Likert questionnaire in each item, which presents the fatigue degree over the past two weeks. CIS includes four proportions: (1) 8 items of subjective fatigue, (2) 4 items of decreased motivation, (3) 3 items of decreased activity, (4) 5 items of decreased concentration. Furthermore, total scale can be calculated by adding the four proportions, the higher the score presents the more serious fatigue status in the four proportions. Beurskens considered CIS can distinguishes of fatigue from the non-fatigue workers effectively [24]. CIS had been applied in plenty investigations in office. Wang et al. had developed Chinese version of CIS with appropriate reliability and validity [25].

Although both well-being and fatigue are important issues in workplace, there is relatively rare investigations of the two issues in workplace, especially assessed by workplace PERMA model and CIS questionnaire.

### Purpose

Hence, our purpose is to establish the scale of workplace well-being, by developing the Chinese version Workplace PERMA-Profiler, and to discovery the association between workplace well-being and fatigue.

## Materials and methods

### Study design

According to the COnsensus-based Standards for the selection of health Measurement INstruments (COSMIN) reporting guidelines [26], we conducted the study and written the manuscript. Each characteristic of the measure was described based on the COSMIN checklists. This was a validation study consisting of baseline and two weeks follow-up surveys in Taiwan throughout January 2021 to August 2021. We used the cross-sectional data to conduct the validity about the Chinese version Workplace PERMA Profiler, including internal consistency, structural validity, as well as convergent validity. Furthermore, we used the two weeks follow-up longitudinal data to analyze the test-retest reliability.

### Participants

Participants were recruited from workers whom receiving health check-up or workplace health service from multi-centers, including the Kaohsiung Medical University Hospital, the Kaohsiung Municipal Siaogang Hospital, and Kaohsiung Municipal Ta-tung Hospital. Participant inclusion criteria were (a) Chinese workers who lived in a city or a county of Taiwan and (b) aged 20 years old or above. Participants with mental disorder previously diagnosed by medical doctor were excluded. Based on these criteria, we recruited workers until the targeted number was reached. The response rate of the participants at the three hospitals were 53.8%, 43.3%, and 34.5% in the Kaohsiung Medical University Hospital, the Kaohsiung Municipal Siaogang Hospital, and Kaohsiung Municipal Ta-tung Hospital, respectively. Of the available respondents, 312 workers met the inclusion criteria and completed the questionnaires. If the eligible workers agreed with the terms and conditions of the questionnaire survey, they could access the self-report questionnaire after informed consent. After two weeks, the randomly sampled 100 participants from the workers who completed the baseline survey again. The study protocol was approved by the Institutional Review Board of Kaohsiung Medical University Hospital, Taiwan [IRB number: KMUHIRB-E(1)-20,200,430]. This study used the G*Power version 3.1.9.236 to calculate the sample size. The minimum effect size for detection in the study was 0.20 (ρ). Finally, the necessary sample size was estimated to be more than 255 in the case of α error probability of 0.05 and power (1 - β) of 0.90, indicating that the participation number was adequate in the investigation.

### Measurements

Participants completed a self-reporting survey, including the Chinese version Workplace PERMA-Profiler, and a questionnaire regarding fatigue, the Checklist Individual Strength (CIS) questionnaire.

### The workplace PERMA-profiler

The multidimensions of workplace well-being was assessed by the Chinese version of the Workplace PERMA-Profiler, including total 23 items rated on an 11-points Likert-type scale (ranging from 0 to 10). In the five factors of PERMA model (Positive emotion, Engagement, Relationships, Meaning, and Accomplishment), each factors contains three items. Furthermore, overall happiness (1 item), negative emotion (3 items), health (3 items), as well as loneliness (1 item) at work were also measured. An average score of the three items in each dimensions consisted of the score of the five PERMA dimensions. An average of the 15 items of PERMA dimensions and Happiness (1 item) was considered as workplace overall happiness. We developed the Chinese version questionnaire based on the International Society of Pharmacoeconomics and Outcomes Research (ISPOR) task force guidelines [27]. First, we contacted and obtained permission from the original developer of the Workplace PERMA-Profiler Dr. Peggy (Margaret) L. Kern to translate the questionnaire into Chinese version. Forward-translation was separately performed and was followed by reconciliation, back-translation, back-translation review, harmonization, and cognitive debriefing. Two experts in Chinese and English affiliated with the Department of Occupational and Environmental Medicine, Kaohsiung Medical University Hospital, who did not know the aim of the study, conducted the back-translation. By means of random sampling, nine Chinese workers, including a company president and occupational health staff members (occupational doctor, public health nurse, clinical psychologist, and human resource management workers), conducted the cognitive debriefing sessions. If the nine workers had difficulty about understanding any sentence in the 23 items, they were invited to accomplish the harmonized measure and revise the wording and to feedback for further revision. Results from the different stages were combined to create the final measure. Please check Appendix 1, the complete of the Chinese version Workplace PERMA-Profiler.

### Checklist individual strength (CIS)

The Chinese version of the checklist individual strength (CIS) was used to determine the participant’s fatigue level [25]. It is a multidimensional questionnaire that consists of 20 items of chronic fatigue measurements. In the collection of participant’s fatigue levels, all participants were asked to indicate how they felt 2 weeks prior to the interview, the result was present in CIS score as a higher CIS score indicates a higher level of fatigue as well as a decrease in concentration, lower the motivation and activity. The whole data collection was completed under a clinical setting [23], furthermore, the total CIS score has shown a 0.88 in Cronbach’s α coefficient, which indicates its validation in the working population [24]. As CIS has covered several aspects of fatigue, this includes the subjective experience of fatigue, reduction in motivation, reduction in activity, and reduction in concentration [28]. The Cronbach’s α of the four aspects of Chinese CIS, Fatigue, Motivation, Concentration, and Physical Activity were 0.84, 0.52, 0.74, and 0.54, respectively. The overall Cronbach’s α coefficient of the Chinese CIS was 0.88, which is within the ideal range [25].

### Statistical analysis

We calculated the statistical values of the Chinese version Workplace PERMA-Profiler, including Cronbach’s alphas, intra-class correlation coefficients (ICC), the standard error of measurement (SEM), and the smallest detectable change (SDC) to test reliability. Furthermore, we also conducted confirmatory factor analysis (CFA) as well as correlational analysis to test validity. We used the statistical software, include SPSS version 21 (IBM) and R 3.5.2 version for statistical analysis.

#### Internal consistency

In accessing the internal consistency, Cronbach’s alphas were used to calculate the Chinese version of the Workplace PERMA-Profiler total score and other factor score (i.e., Positive Emotion, Engagement, Relationships, Meaning, and Accomplishment). According to the current literature [29], higher than 100 is thought as a sufficient sample size for calculating the Cronbach’s alphas. In this study, the Cronbach’s alphas were used in calculating the total score and each factor’s scores according to previous studies [7, 10].

#### Test-retest reliability

The test-retest reliability in the study was evaluated by the Intra-class Correlation Coefficients (ICCs) for the total score and each factor score in a period of two weeks, even though previous studies have shown different parameters (Pearson’s r) as the standard of test-retest reliability, but having a 50–100 participants size for the test-retest reliability analysis was considered perfect [29]. Furthermore, the standards of measurement error were evaluated by the Standard Error of Measurement (SEM) and the Smallest Detectable Change (SDC) [30–32], SME shows the standard deviation of the repeated measures in a single participant. SDC shows the minimal change that a single participant must show on the measurements to ensure that the observed changes are real and not an error in measurement [30]. The SEM was define as (the standard deviation of all testing scores) × √(1 - ICC) [31, 32], and the SDC was defined as 1.96 × √(2 × SEM) [30].

#### Structural validity

In confirming the five-factor structural validity, this study used a CFA to conduct the 15 items by using a robust maximum likelihood estimation in R. In the study several tests were run on the original five-factor model (each of three items was explained by the five factors) and a one-factor model (all 15 items were explained by one factor), this included the chi-square (χ^2^), the Comparative Fit Index (CFI), the Tucker-Lewis Index (TLI), the Root Mean Square Error of Approximation (RMSEA), and the Standardized Root Mean Square Residual (SRMR). The model is considered a perfect fit if the CFI and TLI exceeded 0.95 and the RMSEA and SRMR were less than 0.0635. According to the current literature study [29], the required sample size for factor analysis was five or seven times more than the item numbers, while having a minimum of 100. Given that this Chinese version of the Workplace PERMA-Profiler study has 15 items, a participation number of 105 was considered sufficient.

#### Convergent validity

In the study Pearson’s correlation coefficients (r) are among the PERMA factors that were used to calculate convergent validity, the factors include job and life satisfaction, work engagement, psychological distress, work-related psychosocial factors, and work performance.

## Results

### Demographic characteristics of participants

In the study, 312 participants completed the baseline survey. One hundred workers randomly sampled from the baseline participants in the two weeks follow-up, and the response rate was 65.0% (65 workers) fulfilled the questionnaire without missing values on any items. Table 1 shown the demographic characteristics of the baseline and follow-up workers. In the baseline survey (*N* = 312, 175 men and 137 women, mean age = 40.4 ± 12.2 years), over half of the workers had graduated from university (51.0%). Most participants were full-time workers (91.3%). Over half workers were employed by workplace less than 250 workers (50.3%) and blue-collar workers (61.9%). Compared with the demographic characteristics of the baseline survey, no significant change was observed in the follow-up survey, which 65 participants (37 men and 28 women, mean age 38.8 ± 8.6 years) completed the questionnaire without missing values on any items.

**Table 1:** Demographic characteristics of the participants

	Baseline Survey (*N* = 312)		Follow-up Survey (*N* = 65)	
	n (%)	Mean (SD)	n (%)	Mean (SD)
Gender				
Male	175 (56.1%)		37 (56.9%)	
Female	137 (43.9%)		28 (43.1%)	
Age		40.4 (12.2)		38.8 (8.6)
Educational status				
Below university	153 (49.0%)		30 (46.2%)	
University	159 (51.0%)		35 (53.8%)	
Employ status				
Full-time	285 (91.3%)		61 (93.8%)	
Non full-time	27 (8.7%)		4 (6.2%)	
Shift status				
Day shift	249 (79.8%)		56 (86.2%)	
Shift work	63 (20.2%)		9 (13.8%)	
Job type				
White collar	119 (38.1%)		25(38.5%)	
Blue collar	193 (61.9%)		40(61.5%)	
Size of workplace				
<250 employees	157 (50.3%)		33 (50.8%)	
≥250 employees	155 (49.7%)		32 (49.2%)	

### The validity and reliability of Chinese workplace PERMA profiler

#### Internal consistency and test-retest reliability

Table 2 demonstrated mean scores, Cronbach’s alphas (α), ICCs, SEMs, and SDCs for the Workplace PERMA factors. Cronbach’s alpha coefficients ranged from 0.69 to 0.93. ICCs ranged from 0.70 to 0.92, meaning that approximately 80% of variance in two-time measurements was explained by individuals. SDCs ranged from 0.47 to 0.89.

**Table 2:** Mean scores, internal consistency, and reliability of the Chinese version Workplace PERMA-Profiler (*N* = 312)

Factors	Baseline Mean (SD)	Min-Max	Cronbach’s alpha	Follow-up Mean (SD) †	Test-retest †	SME †	SDC †
Positive emotion	6.5 (1.7)	1–10	0.83	7.0 (1.6)	0.87**	0.20	0.57
P1	6.2 (2.0)	0–10		6.8 (2.0)	0.81**	0.25	0.69
P2	7.1 (1.9)	2–10		7.4 (1.6)	0.78**	0.20	0.56
P3	6.3 (2.0)	0–10		6.7 (2.0)	0.83**	0.25	0.68
Engagement	6.6 (1.7)	2–10	0.78	7.0 (1.7)	0.90**	0.21	0.57
E1	6.9 (1.9)	0–10		7.2 (1.9)	0.84**	0.23	0.64
E2	6.6 (2.0)	0–10		6.8 (2.1)	0.81**	0.26	0.72
E3	6.3 (2.3)	0–10		6.9 (2.0)	0.84*	0.25	0.70
Relationships	6.6 (1.6)	1–10	0.72	6.7 (1.7)	0.90**	0.21	0.59
R1	6.9 (2.1)	0–10		7.1 (2.3)	0.76**	0.28	0.78
R2	5.9 (2.0)	0–10		6.0 (2.2)	0.79**	0.28	0.76
R3	7.0 (1.8)	1–10		7.0 (2.0)	0.77**	0.25	0.70
Meaning	6.9 (1.7)	0.3–10	0.86	7.1 (1.6)	0.92**	0.20	0.56
M1	6.9 (1.9)	1–10		7.0 (1.9)	0.82**	0.24	0.67
M2	6.8 (1.9)	0–10		7.1 (1.7)	0.84**	0.22	0.60
M3	6.9 (1.8)	0–10		7.0 (1.9)	0.85**	0.23	0.65
Accomplishment	7.2 (1.5)	2.7–10	0.69	7.6 (1.4)	0.87**	0.17	0.47
A1	6.9 (1.9)	0–10		7.2 (2.1)	0.86**	0.26	0.73
A2	7.1 (1.9)	0–10		7.4 (1.7)	0.84**	0.21	0.58
A3	7.7 (1.7)	0–10		8.1 (1.5)	0.78**	0.19	0.52
Happiness	6.5 (2.0)	0–10		6.8 (1.8)	0.76**	0.26	0.72
Overall well-being (16 items)	6.8 (1.4)	1.9–10	0.93	7.1 (1.4)	0.92**	0.17	0.48
Negative emotion (3 items)	3.7 (2.1)	0–10	0.83	3.9 (2.0)	0.83**	0.25	0.69
Health (3 items)	6.2 (2.0)	0–10	0.93	6.8 (1.8)	0.90**	0.22	0.61
Loneliness (1 item)	3.7 (2.5)	0–10		4.0 (2.6)	0.70**	0.32	0.89

† *N* = 65. ICC: intra-class correlation coefficient, SME: standard error of measurement, SDC: smallest detectable change. ** p value < 0.01

#### Structural validity

**Fig. 1:**
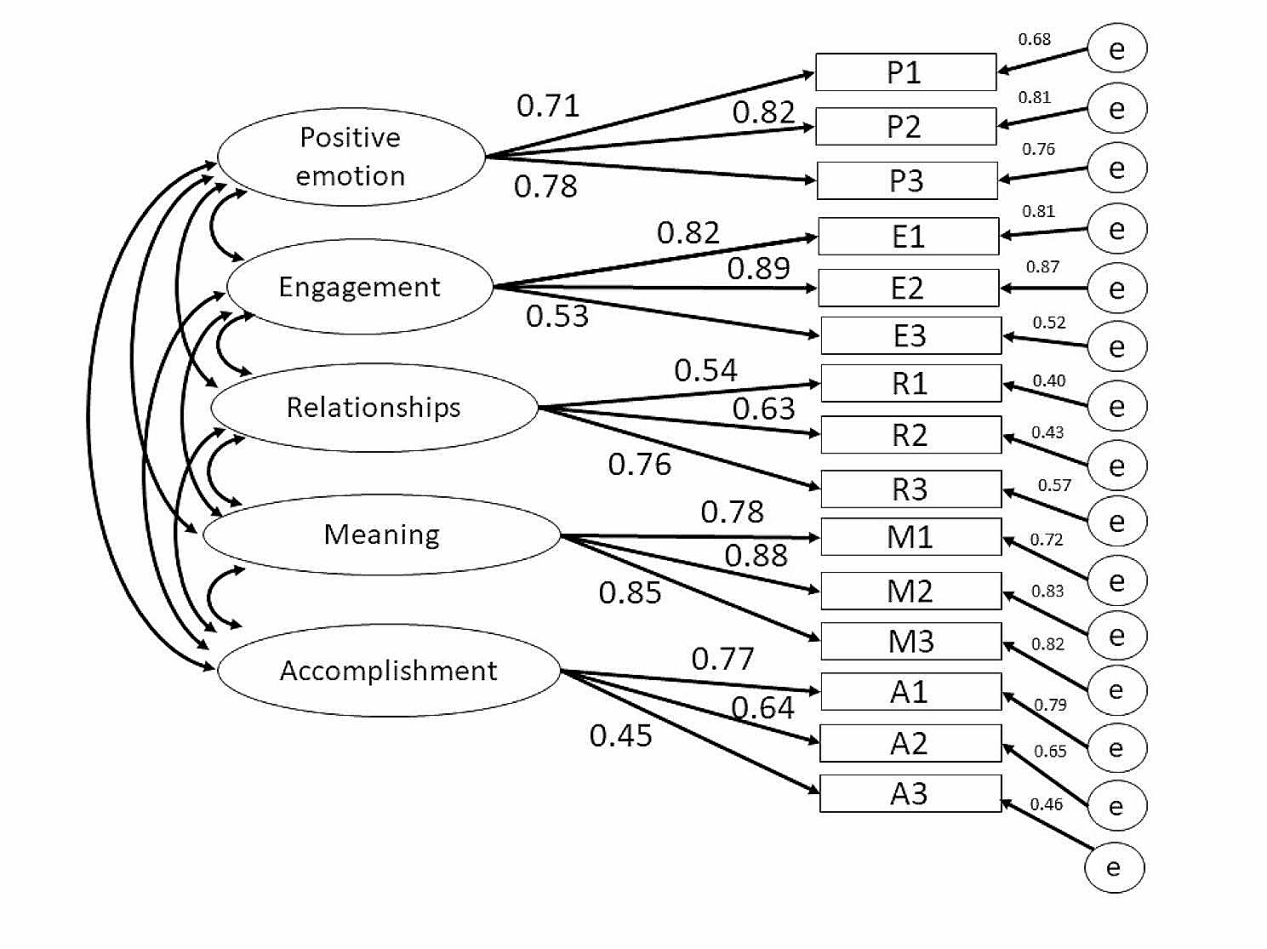
The diagram of the confirmatory factor analysis (CFA) for the Workplace PERMA Profiler

Table 3; Fig. 1 revealed the results of CFA. Standardized covariances ranged from 0.453 to 0.890 in the 5-factors model, implied a strong correlation. The five-factor model shown a nearly appropriate fit (χ^2^ [82] = 346.56, CFI = 0.887, TLI = 0.855, RMSEA = 0.114, SRMR = 0.06), which demonstrated a better fit (Δχ^2^ [8] = 141.688, *p* < 0.05) compared with one-factor model.

**Table 3:** Factor loadings of the 15 Workplace PERMA Profiler items, factor correlations, and model fit in confirmatory factor analyses

Items	Factor loadings			Correlation coefficients in the 5-factor model					
	1-factor model	5-factor model							
P1	0.683*	0.712*			F1 (P)	F2 (E)	F3 (R)	F4 (M)	F5 (A)
P2	0.814*	0.819*		F1 (P)	1.000				
P3	0.761*	0.775*		F2 (E)	1.000^†^	1.000			
E1	0.808*	0.817*		F3 (R)	0.815*	0.643*	1.000		
E2	0.865*	0.890*		F4 (M)	0.862*	0.910*	0.606*	1.000	
E3	0.522*	0.528*		F5 (A)	0.994*	0.962*	0.834*	1.000^†^	1.000
R1	0.403*	0.542*		Model fit		1-factor		5-factor	
R2	0.432*	0.627*		X^2^		488.248 (90) *		346.560 (82) *	
R3	0.573*	0.755*		CFI		0.829		0.887	
M1	0.718*	0.778*		TLI		0.801		0.855	
M2	0.828*	0.875*		RMSEA (95% CI)		0.134 (0.122, 0.146) *		0.114 (0.102, 0.127) *	
M3	0.817*	0.849*		SRMR		0.074		0.060	
A1	0.791*	0.774*		1-factor model vs. 5-factor model: Δχ^2^ (df) 141.688 (8) *					
A2	0.649*	0.636*							
A3	0.457*	0.453*							

The robust maximum likelihood estimation method was used. *p value < 0.05

^†^ The correlations between F1 (P) and F2 (E), and F4 (M) and F5 (A) in the 5-factor model were constrained to be 1.00 because a free estimation had fallen proper solution

#### Convergent validity

Table 4 declared Pearson’s correlation coefficients (γ) among the Workplace PERMA factors, and CIS. The five Workplace PERMA factors and Happiness had small to moderate negative correlations with CIS ( -0.595 ≤ γ≤ -0.309). Furthermore, they had small to moderate negative correlations with the dimensions of Fatigue, Concentration, and Motivation in CIS ( -0.606 ≤ γ≤ -0.233). Only Physical Activity had relatively weak associations (-0.220 ≤ γ≤ -0.099).

**Table 4:** Convergent validity (r) of the Chinese version of the Workplace PERMA-profiler (*N* = 312)

Variables	P	E	R	M	A	Hap
Workplace PERMA Profiler						
Positive emotion (P)	1.000					
Engagement (E)	0.780**	1.000				
Relationships (R)	0.626^**^	0.489^**^	1.000			
Meaning (M)	0.676^**^	0.744^**^	0.481^**^	1.000		
Accomplishment (A)	0.656^**^	0.648^**^	0.545^**^	0.719^**^	1.000	
Happiness (Hap)	0.855**	0.708^**^	0.589^**^	0.589^**^	0.548^**^	1.000
CIS	-0.595^**^	-0.366^**^	-0.309^**^	-0.338^**^	-0.348^**^	-0.535^**^
Fatigue (CIS)	-0.511^**^	-0.263^**^	-0.276^**^	-0.250^**^	-0.233^**^	-0.481^**^
Concentration (CIS)	-0.545^**^	-0.354^**^	-0.260^**^	-0.314^**^	-0.342^**^	-0.482^**^
Motivation (CIS)	-0.606^**^	-0.517^**^	-0.338^**^	-0.431^**^	-0.432^**^	-0.546^**^
Physical activity (CIS)	-0.292^**^	-0.099	-0.179^**^	-0.109	-0.213^**^	-0.220^**^

**p < 0.01

### The association between of workplace PERMA profiler and CIS

Table 5 shown the regression model of the CIS according to the PERMA dimensions and happiness of Workplace PERMA-Profiler, adjusting for co-founding factors. Age and Positive Emotion were significantly and negatively correlated with the total of CIS (β-values − 0.169, and − 0.7; p-values 0.003, and < 0.001, respectively). Engagement was significantly and positively correlated with the total of CIS (β-values 0.303; p-value 0.001). Age, Positive Emotion, and Happiness were significantly and negatively correlated with Fatigue (β-values − 0.191, -0.657 and − 0.199; p-values 0.001, < 0.001, and 0.040, respectively). Engagement was significantly and positively correlated with Fatigue (β-values 0.393; p-value < 0.001). Age and Positive Emotion were significantly and negatively correlated with Concentration (β-values − 0.148, and − 0.634; p-values 0.013, and < 0.001, respectively). Engagement was significantly and positively correlated with Concentration (β-values 0.211; p-values 0.02). Positive Emotion was significantly and negatively correlated with Motivation (β-values − 0.443; p-values < 0.001). Positive Emotion and Accomplishment were significantly and negatively correlated with Physical Fatigue (β-values − 0.531, and − 0.185; p-values < 0.001, and 0.03, respectively).

**Table 5:** Regression model of CIS according to the PERMA dimensions of Workplace PERMA-Profiler.

	CIS						Fatigue						Concentration			
	B		SE	β	*P* value		B	SE	β	*P* value			B	SE	β	*P* value
Age (years)	-0.248		0.083	-0.169	0.003*		-0.147	0.045	-0.191	0.001*			-0.061	0.024	-0.148	0.013*
Gender (male)	0.141		1.706	0.004	0.934		0.137	0.941	0.007	0.884			-0.047	0.506	-0.005	0.926
Education (university and above)	0.566		1.960	0.016	0.773		-0.929	1.073	-0.050	0.387			0.555	0.581	0.055	0.340
Employment status (full-time)	0.183		2.918	0.003	0.950		0.650	1.612	0.020	0.687			-0.522	0.865	-0.029	0.547
Shift status (shift work)	-1.956		2.199	-0.044	0.374		-0.803	1.210	-0.035	0.507			-0.735	0.652	-0.058	0.260
Occupation (white collar)	-0.759		1.883	-0.021	0.687		-0.787	1.038	-0.041	0.449			0.081	0.558	0.008	0.885
Size of worksite (≥ 250 employees)	0.601		1.836	0.017	0.744		-0.079	1.013	-0.004	0.938			0.136	0.544	0.013	0.803
Positive Emotion	-7.375		1.103	-0.700	< 0.001*		-3.615	0.609	-0.657	< 0.001*			-1.888	0.327	-0.634	< 0.001*
Engagement	3.187		0.906	0.303	0.001*		2.166	0.501	0.393	< 0.001*			0.626	0.269	0.211	0.020*
Relationship	0.799		0.710	0.070	0.262		0.234	0.389	0.039	0.548			0.337	0.211	0.104	0.111
Meaning	0.050		0.855	0.005	0.953		-0.061	0.469	-0.011	0.898			0.110	0.253	0.036	0.664
Accomplishment	-0.650		0.870	-0.053	0.456		0.287	0.480	0.045	0.551			-0.350	0.258	-0.101	0.176
Happiness	-1.126		0.808	-0.127	0.165		-0.916	0.444	-0.199	0.040*			-0.189	0.240	-0.075	0.432
		Motivation					Physical									
		B	SE	β	p value		B	SE	β		p value					
Age (years)		-0.033	0.019	-0.099	0.081		-0.014	0.014	-0.065		0.339					
Gender (male)		-0.132	0.392	-0.016	0.736		0.101	0.300	0.019		0.736					
Education (above university)		0.245	0.447	0.030	0.584		0.431	0.342	0.082		0.208					
Employment status (full-time)		-0.487	0.671	-0.034	0.469		0.572	0.513	0.062		0.266					
Shift status (shift work)		-0.654	0.504	-0.065	0.196		0.432	0.385	0.066		0.264					
Occupation (white collar)		0.113	0.432	0.014	0.793		-0.034	0.330	-0.006		0.919					
Size of worksite (≥ 250 employees)		0.671	0.422	0.083	0.113		-0.026	0.323	-0.005		0.935					
Positive Emotion		-1.059	0.254	-0.443	< 0.001*		-0.816	0.194	-0.531		< 0.001*					
Engagement		-0.151	0.208	-0.063	0.468		0.565	0.159	0.367		< 0.001*					
Relationship		0.169	0.162	0.065	0.299		-0.026	0.124	-0.016		0.833					
Meaning		-0.013	0.196	-0.005	0.949		0.102	0.150	0.065		0.496					
Accomplishment		-0.215	0.200	-0.077	0.283		-0.333	0.153	-0.185		0.030*					
Happiness		-0.195	0.185	-0.097	0.292		0.089	0.141	0.069		0.529					

CIS, Checklist Individual Strength; *p-value < 0.05

## Discussion

From our study, the Chinese Workplace PERMA-Profiler revealed good reliability, convergent validity, as well as adequate structural validity. It shown a good internal consistency and was mostly steady over two-weeks period. In the Chinese version Workplace PERMA-Profiler, profound differences in workplace wellbeing could be identified around 1 point within the 11-point Likert scale. This questionnaire could be useful for evaluation of workplace well-being and intervention studies among Chinese workers in the future. The Workplace PERMA Profiler is a specific adaptation of Seligman’s PERMA model, designed to assess well-being within the workplace context by Dr. Kern in 2016. Seligman’s PERMA model, which stands for Positive Emotion, Engagement, Relationships, Meaning, and Achievement, provides a broad framework for understanding the components of well-being. The Workplace PERMA Profiler utilizes this framework but focuses on aspects particularly relevant to the work environment. While Seligman’s five dimensions of well-being provide a foundational framework, the Workplace PERMA Profiler adapts and refines this model to make it directly applicable and beneficial in a work context. The Workplace PERMA Profiler, building on Seligman’s foundational PERMA model, enriches the original by adding 8 optional items to the core 15, aiming for a more nuanced evaluation of workplace well-being by covering aspects like happiness, negative emotions, health, and loneliness. Incorporating a variety of constructs raises valid concerns about the scale’s factorial and content validity, yet it seeks to provide a holistic view of employee well-being that transcends basic PERMA elements. The profiler’s translation into multiple languages, including Japanese, Korean, and Chinese, attests to its versatility and ensure it accurately captures well-being across different cultures.

### CFA

The CFA demonstrated that the 5-factor Workplace-PERMA model had a nearly appropriate fit [χ^2^ (82) = 346.56, CFI = 0.887, TLI = 0.855, RMSEA = 0.114, SRMR = 0.060], instead of completely supporting the 5-factor model of the measure. Nevertheless, the original PERMA Profilers (CFI = 0.894, TLI = 0.864, RMSEA = 0.107) [10], Japanese version (CFI = 0.892, TLI = 0.858, RMSEA = 0.105) [33] and Korean version (CFI = 0.909, TLI = 0.881, RMSEA = 0.110) [34] of the Workplace PERMA Profiler shown the similar results. The nearly appropriate fit implies that these items might not sufficiently distinguish the 5 factors in the Chinese version, or the 5-factor model might not be the most suitable model for assessing workplace well-being. Previous Chinese version of wellbeing models or workplace wellbeing models have concentrated on life satisfaction, positive affect and negative affect [35] or its association between work healthy promoting lifestyle and work environmental satisfaction, based on two constructs (contentment and joyfulness) [36]. Although the PERMA model divides well-being as five dimensions may not be a beneficial distinction in the workplace, these dimensions provide more specific concepts than the other broader dimensions, such as life satisfaction or work environment satisfaction [37]. Further studies are required to explore how Chinese workers realize each item in the five dimensions by means of qualitative research (e.g., focus group interviews or other methods), or integrate PERMA and other well-being models into a more appropriate version for general population of Chinese workers.

### Convergent validity

Convergent validity was also well established in our study. The effect sizes for five dimensions of Workplace PERMA-Profiler and Happiness were similar with several previous validation studies [10, 33, 34]. Moreover, CIS revealed the negative correlations with the measurement of PERMA five dimensions and Happiness, especially with the Workplace PERMA-P (Positive Emotion) dimension. The association with Workplace PERMA-P dimension were also similar with the subscales of CIS, including Subjective Fatigue, Concentration, Motivation, and Physical Activity dimensions. On the contrary, Adriaenssens et al. found a positive correlation between prolonged fatigue and psycho-somatic distress [38]. The findings of these studies were consistent with the trend of our results. Sooner or later, further studies may be considered to investigate the extent to which tool of the well-being assessment can foresee future fatigue at workplace.

### Workplace Well-being and fatigue: workplace PERMA-profiler and CIS

To our knowledge, this is the first study about the association between fatigue and well-being in workplace, assessed by CIS and Workplace PERMA-Profiler. We found the hedonic well-being (Positive Emotion, Engagement, and Happiness) play more important roles in fatigue than the eudaimonic well-being (Meaning and Accomplishment). Similar results had been demonstrated in Moreno et al.’s study, which shown fatigue was associated with hedonic wellbeing component rather than eudaimonic well-being [39]. Compared with hedonic well-being, eudaimonic well-being is not just characterized by being free from these behavioral symptoms, including fatigue. Hedonic well-being (Positive Emotion, Engagement, and Happiness) plays a more significant role in fatigue than eudaimonic well-being (Meaning and Accomplishment) possibly due to the fundamental differences between these two dimensions of well-being. Hedonic well-being is centered on the pursuit of pleasure, happiness, and the avoidance of pain. It emphasizes immediate feelings of joy, satisfaction, and comfort. In contrast, eudaimonic well-being focuses on meaning, self-realization, and fulfilling one’s potential, which are more abstract and long-term goals. The pursuit of hedonic well-being can lead to immediate psychological and physiological benefits, such as reduced stress levels, improved mood, and increased energy levels, directly counteracting feelings of fatigue. While eudaimonic pursuits are beneficial for long-term mental health and resilience, they may not offer the same direct impact on daily experiences of fatigue. Although both dimensions of well-being are crucial for overall health, hedonic well-being has a more direct and immediate role in managing fatigue due to its focus on pleasure, comfort, and the alleviation of discomfort. About the relationship and fatigue, Wada et al. investigated the association between working conditions and prolonged fatigue among Japanese physicians, revealing that both workload and career satisfaction significantly impact fatigue levels. Male physicians’ fatigue was linked to their relationships with colleagues and staff, whereas female physicians’ fatigue correlated with the amount of personal time. The findings suggest the importance of considering workload, career satisfaction, interpersonal relationships, and personal time in managing physician fatigue in Japan [40]. Watterson and colleagues found a link between fatigue and pharmacists’ interpersonal relationships, including those with family, team members, and patients in US. Mental fatigue manifests as a lack of motivation—characterized by feelings of being uninterested, indifferent, passive, listless, and lacking initiative—and sleepiness. Participants identified their fatigue through changes in their interactions with staff members and patients, noting, for instance, an increase in irritability and crankiness with customers as the day progressed [41]. In contrast, our study did not find a negative association between fatigue and relationship. This discrepancy could stem from differences in the study populations’ careers or cultural contexts, suggesting that the impact of fatigue on interpersonal relationships may vary significantly across professions and cultural settings. While Wada’s and Watterson’s studies point to a clear link between fatigue and the quality of professional and personal interactions, our findings suggest that this relationship might not be as straightforward or might manifest differently, highlighting the need for a nuanced understanding of fatigue’s impacts across different cultural and professional landscapes. On the other hand, our study shown Positive Emotion was significantly negatively associated with CIS and its four scales, while Engagement was almost significantly positively associated with CIS and its three subscales, expect Motivation. Although the causal relation between the hedonic well-being components and long-term fatigue cannot be well-established in our study, several prior studies may provide some clues. Lack of psychological disengagement from work is a major mechanism by which job strain turn into fatigue [42, 43]. Yamada et al. found improvement in fatigue was a significant and positive predictor of occupational re‑engagement [44].

There are several limitations in the study. First, we recruited workers fulfill the questionnaire until reaching the targeted number and no random sampling was conducted, selection bias might occur. For example, workers who were more well-being and less fatigue might be more willing to complete the survey. Second, the assessment of the standards of convergent validity might have measurement errors. Third, other potential co-variants not measured might misrepresent the association of correlation, for example psychological capital or organizational culture. Fourth, because no random sampling study design, the generalizability and application of the results for all population of Chinese-speaking workers might be doubted. Fifth, the causal relation between well-being and fatigue cannot be declared in the cross-sectional survey, further studies will be required for establishing the causal relation and possible mechanism.

## Conclusion

In the study, the Chinese version Workplace PERMA-Profiler shown good reliability and validity. This questionnaire could be practical to evaluate well-being in workplace, advocate workplace well-being investigation for Chinese-speaking workers, and provide a solution of defining well-being in further research. Furthermore, about the association between chronic fatigue and Workplace PERMA-Profiler, the CIS and its four dimensions were significantly negative associated with the Positive Emotion, while they are significantly positive associated with Engagement dimension except CIS-Motivation dimension. CIS-Physical activity dimension was significantly negatively associated with Accomplishment.

### Electronic supplementary material

Below is the link to the electronic supplementary material.

Supplementary Material 1

## Data Availability

The original contributions presented in the study are included in the article/supplementary material, further inquiries can be directed to the corresponding author.

## Appendix

Chinese version Workplace PERMA-Profiler.

## Abbreviations

CFA: Confirmatory factor analysis
CFI: Comparative Fit Index
CIS: Checklist Individual Strength
COSMIN: COnsensus-based Standards for the selection of health Measurement INstruments
ICC: Intra-class Correlation Coefficients
ISPOR: International Society of Pharmacoeconomics and Outcomes Research
PERMA: Positive Emotion, Engagement, Relationship, Meaning, Accomplishment
RMSEA: Root Mean Square Error of Approximation
SDC: Smallest Detectable Change
SEM: Standard Error of Measurement
SRMR: Standardized Root Mean Square Residual
TLI: Tucker-Lewis Index
