# Supplementary material for: The validation of Chinese version of workplace PERMA-profiler and the association between workplace well-being and fatigue
Source: BMC Public Health. 2024 Mar 6;24:720. doi: 10.1186/s12889-024-18194-6 (PMC10916278; doi:10.1186/s12889-024-18194-6)
Supplement: Supplementary file 1 — Supplementary Material 1 [file 12889_2024_18194_MOESM1_ESM.pdf]

Chinese version Workplace PERMA Profiler 中文版職場 PERMA 量表

For English version of questionnaire please refer to this link: https://www.peggykern.org/uploads/5/6/6/7/56678211/workplace_perma_profiler_102014.pdf

請閱讀以下編號 1～23 的句子，從「0」至「10」之中選出一個最貼切的數字，並參照回答範例進行圈選。	
回答範例	**0%** **100%**<br>0 1 2 3 4 (5) 6 7 8 9 10
1	您的工作具備目的性、意義性的程度為何？<br>**0%** **100%**<br>0 1 2 3 4 5 6 7 8 9 10
2	您有多常認為自己正朝著工作上的目標邁進？<br>**完全不認為** **總是這麼認為**<br>0 1 2 3 4 5 6 7 8 9 10
3	在職場上，您有多常埋頭熱衷於自己的工作中？<br>**完全沒有** **總是如此**<br>0 1 2 3 4 5 6 7 8 9 10
4	一般而言，您覺得自己的健康狀態如何？<br>**非常差** **非常好**<br>0 1 2 3 4 5 6 7 8 9 10
5	在職場上，您有多常感到快樂？<br>**完全不認為** **總是這麼認為**<br>0 1 2 3 4 5 6 7 8 9 10
6	在需要時，您有多常得到職場同仁的幫助或配合？<br>**完全沒有** **總是如此**<br>0 1 2 3 4 5 6 7 8 9 10
7	在職場上，您有多常感到不安？<br>**完全不認為** **總是這麼認為**<br>0 1 2 3 4 5 6 7 8 9 10

8	您有多常達成自己在工作上設立的重要目標？<br>**完全無法達成** **總是能夠達成**<br>0 1 2 3 4 5 6 7 8 9 10
9	一般而言，您有多常認為自己在職場上從事的工作，屬於重要且有價值之事？<br>**完全不認為** **總是這麼認為**<br>0 1 2 3 4 5 6 7 8 9 10
10	在職場上，您有多常抱持正向（積極）心態？<br>**完全不認為** **總是這麼認為**<br>0 1 2 3 4 5 6 7 8 9 10
11	您對自己的工作抱持興奮、興趣的程度為何？<br>**0%** **100%**<br>0 1 2 3 4 5 6 7 8 9 10
12	您有多常在職場上感到孤獨？<br>**完全不認為** **總是這麼認為**<br>0 1 2 3 4 5 6 7 8 9 10
13	您對目前健康狀態的滿意程度為何？<br>**0%** **100%**<br>0 1 2 3 4 5 6 7 8 9 10
14	在職場上，您有多常感到憤怒？<br>**完全不認為** **總是這麼認為**<br>0 1 2 3 4 5 6 7 8 9 10
15	您有多常認為自己受到職場同仁的感謝？<br>**完全不認為** **總是這麼認為**<br>0 1 2 3 4 5 6 7 8 9 10
16	您有多常完成在工作上的責任？<br>**完全無法完成** **總是能夠完成**<br>0 1 2 3 4 5 6 7 8 9 10

17	一般而言，您覺得工作上具備方向性的程度為何？<br>**0%** ... **100%**<br>0 1 2 3 4 5 6 7 8 9 10
18	與同年齡層且同性別的人們相比，您的健康狀態如何？<br>**非常差** ... **非常好**<br>0 1 2 3 4 5 6 7 8 9 10
19	您對自己在職務上的人際關係滿意程度為何？<br>**完全不滿意** ... **十分滿意**<br>0 1 2 3 4 5 6 7 8 9 10
20	在職場上，您有多常感到悲傷？<br>**完全不認為** ... **總是這麼認為**<br>0 1 2 3 4 5 6 7 8 9 10
21	當您樂在工作之中，有多常忘記時間的流逝？<br>**完全沒有** ... **總是如此**<br>0 1 2 3 4 5 6 7 8 9 10
22	在職場上，您有多常感到滿足？<br>**完全不認為** ... **總是這麼認為**<br>0 1 2 3 4 5 6 7 8 9 10
23	綜合所有要素，您覺得目前從事的工作帶來的幸福程度為何？<br>**0%** ... **100%**<br>0 1 2 3 4 5 6 7 8 9 10

Chen-Cheng Yang et al.

abcmacoto@gmail.com
